# Quantitation of PET spatial extent as a potential adjunct to visual interpretation of [^18^F]flortaucipir imaging: TAU-SPEX

**DOI:** 10.1007/s00259-025-07384-y

**Published:** 2025-06-07

**Authors:** Emma M. Coomans, Bastiaan van Tol, Colin Groot, Ruben Smith, Sebastian Palmqvist, Erik Stomrud, Michael J. Pontecorvo, Sergey Shcherbinin, Ian Kennedy, Vikas Kotari, Wiesje M. van der Flier, Yolande A. L. Pijnenburg, Niklas Mattsson-Carlgren, Oskar Hansson, Elsmarieke van de Giessen, Rik Ossenkoppele

**Affiliations:** 1 Alzheimer Center Amsterdam, Neurology, Vrije Universiteit Amsterdam, Amsterdam UMC location VUmc, Amsterdam, The Netherlands; 2 Amsterdam Neuroscience, Neurodegeneration, Amsterdam, The Netherlands; 3 Clinical Memory Research Unit, Department of Clinical Sciences in Malmö, Lund University, Lund, Sweden; 4 Memory Clinic, Skåne University Hospital, Malmö, Sweden; 5 Eli Lilly and Company, Indianapolis, IN USA; 6 Wallenberg Center for Molecular Medicine, Lund University, Lund, Sweden; 7 Radiology & Nuclear Medicine, Vrije Universiteit Amsterdam, Amsterdam UMC location VUmc, Amsterdam, The Netherlands; 8 Amsterdam Neuroscience, Brain Imaging, Amsterdam, The Netherlands

**Keywords:** Tau, PET, Alzheimer’s disease, Diagnosis

## Abstract

**Purpose:**

Among visually Tau-PET-positive scans, large variation exists in the size of the visually tau-positive area. Here, we propose a metric quantifying the spatial extent of visual Tau-PET-positivity, termed “TAU-SPEX”, and evaluate associations with visual read status, neurofibrillary tangle (NFT) pathology at autopsy, and cognition.

**Methods:**

[^18^F]flortaucipir data from 1,645 participants (aged 71.9 ± 8.2 years, 50.3% females) from four cohorts were visually read as positive or negative. TAU-SPEX was calculated as the percentage of gray matter voxels with suprathreshold Tau-PET uptake (using a threshold identical to that used for visual reading) in a spatially unconstrained whole-brain mask. We additionally computed Tau-PET SUVr in a whole-brain and temporal meta-region. We tested the performance of TAU-SPEX for distinguishing visually tau-negative from tau-positive participants and for distinguishing participants with and without NFT Braak-V/VI pathology at autopsy (*n* = 18), and tested associations of TAU-SPEX with concurrent and longitudinal cognition. The performance of TAU-SPEX was compared to SUVr.

**Results:**

TAU-SPEX demonstrated strong performance in distinguishing tau-negative from tau-positive participants (AUC: 0.97). Moreover, TAU-SPEX showed high accuracy, sensitivity, specificity, positive predictive value and negative predictive value (all > 0.90) for identifying tau-positive participants, and showed high sensitivity (87.5%) and specificity (100.0%) for identifying participants with NFT Braak-V/VI pathology. TAU-SPEX was moderately associated with concurrent (β=-0.36 [-0.29, -0.43], *p* < 0.001) and longitudinal (β=-0.19 [-0.15, -0.22], *p* < 0.001) cognition. Across analyses, TAU-SPEX generally outperformed SUVr.

**Conclusion:**

TAU-SPEX was strongly associated with visual read, NFT Braak-V/VI pathology and cognition, and might be useful in clinical settings as a potential adjunct to [^18^F]flortaucipir visual interpretation.

**Supplementary Information:**

The online version contains supplementary material available at 10.1007/s00259-025-07384-y.

## Introduction

Tau neurofibrillary tangles (NFT) are a key pathological hallmark of Alzheimer’s disease, and can be visualized and quantified during life using PET. Tau-PET is an accurate diagnostic tool for distinguishing Alzheimer’s disease dementia from other dementias [1–4], and is strongly correlated with cognitive decline, clinical progression, and brain atrophy [5–10]. A visual read method for the Tau-PET radiotracer [^18^F]flortaucipir was approved by the US Food and Drug Administration (FDA) (in 2020) and the European Medicines Agency (EMA) (in 2024) to support the clinical diagnosis of symptomatic Alzheimer’s disease [11]. However, among individuals with a positive Tau-PET visual read, there is significant variation in the size of the tau-positive area, which may provide valuable diagnostic and prognostic information. Hence, while visual reads facilitate the clinical implementation of Tau-PET for diagnostic purposes, its dichotomous nature (i.e. positive/negative) does not fully capitalize on the full diagnostic, prognostic and disease monitoring potential of Tau-PET.

A recent survey study among dementia and PET experts worldwide showed that 81% of respondents envision that Tau-PET in clinic should be assessed by combining visual reads with quantitative metrics. Similarly, 63.5% of respondents believed this combined approach should be applied in trials [12]. Tau-PET is most commonly quantified using standardized uptake value ratio (SUVr) in a temporal meta-region-of-interest (ROI) [13], which can be interpreted as a measure of regional tau burden. However, Tau-PET SUVr has several limitations. First, quantifying SUVr in a temporal meta-ROI essentially applies a one-size-fits-all approach and ignores the possibility that tau outside this region may also affect clinical trajectories. Moreover, since SUVr averages signal across a target region, it underutilizes the detailed spatial information provided by PET (as scans with focal high-intensity tracer uptake can result in identical SUVr values as scans with more widespread average-intensity tracer uptake). Second, [^18^F]flortaucipir SUVr demonstrates a substantial amount of variance among participants expected to have limited NFT pathology in neocortical regions, such as in amyloid-negative cognitively unimpaired participants, possibly reflecting off-target binding [14]. Third, in a clinical context, SUVr values are relatively challenging to interpret for patients and clinicians due to the lack of intuitive benchmarks and reference standards. These limitations may be overcome by quantifying the spatial extent of tau-tracer signal rather than the intensity of tau-tracer signal [15–18], which can be operationalized as the percentage of grey matter brain tissue showing suprathreshold Tau-PET uptake. Spatial extent is a spatially unconstrained metric that is not limited to an a-priori defined ROI, as it counts every voxel with suprathreshold Tau-PET uptake– also those occurring outside of the temporal meta-ROI or in relatively small or unilateral clusters (which would mostly be diluted in an SUVr metric). Moreover, spatial extent binarizes each voxel, and may thus be less affected by subthreshold noise-related variation in Tau-PET uptake. Finally, spatial extent is more intuitive to interpret, as it has a well-defined starting point (0%) and ceiling (100%).

Given the recent regulatory approvals for Tau-PET to be used clinically, the interest from the field to combine visual read with quantitative metrics [12], and the potential advantages of spatial extent as compared to SUVr, we aimed to develop a voxel-wise Tau-PET spatial extent metric (hereafter referred to as “TAU-SPEX”) that aligns with the [^18^F]flortaucipir visual read framework and captures interindividual variability in the size of the visually tau-positive area. Specifically, using [^18^F]flortaucipir PET data from 1,645 participants, we aimed to (i) characterize TAU-SPEX among visually tau-negative and visually tau-positive individuals, (ii) assess concordance of TAU-SPEX with Tau-PET visual read, (iii) compare antemortem TAU-SPEX to postmortem NFT pathology in advanced Braak stages (i.e. Braak stages V-VI) at autopsy, and (iv) investigate the relationship between TAU-SPEX with concurrent and longitudinal cognitive functioning in visual read tau-positive individuals. For comparison purposes, analyses were additionally performed for SUVr in a spatially unconstrained whole-brain ROI (similarly to TAU-SPEX) as well as for SUVr in the commonly used temporal meta-ROI [19]. 

## Methods

### Participants

We included participants from the Amsterdam Dementia Cohort (ADC, *n* = 181) [20], the Swedish BioFINDER-1 study (*n* = 238) (NCT01208675), a cohort including multiple studies from Eli Lilly (*n* = 485, consisting of participants in the A04 [NCT02051764, *n* = 36], A05 [NCT02016560, *n* = 200], A08 [NCT04468347, *n* = 76], placebo arm of the Eli Lilly solanezumab Expedition-3 trial [NCT01900665, *n* = 89], and placebo arm of the Eli Lilly LY3202626 NAVIGATE-AD trial [NCT02791191, *n* = 84] studies), and the Alzheimer’s Disease Neuroimaging Initiative (ADNI, *n* = 741) [21]. Characteristics of each cohort are described in Supplementary Table 1. Written informed consent was obtained from all participants or their assigned surrogate decision-makers prior to cohort enrollment. Ethical approval for all cohorts included in the current study was obtained from local institutional review boards.

Participants were required to be aged 50 or older, and to have a suitable Tau-PET scan available to obtain visual read, SUVr and TAU-SPEX. Cognitively unimpaired participants had to have neuropsychological test scores within the normative range. Participants with MCI were defined as having a decline in memory or another cognitive domain objectively verified by neuropsychological testing, while not meeting criteria for dementia and having no or minimal impairment in activities of daily living [22]. Participants with a syndromic dementia diagnosis met diagnostic criteria for Alzheimer’s disease-type dementia (*n* = 453, among which *n* = 5 primary progressive aphasia, *n* = 5 posterior cortical atrophy, and *n* = 2 behavioral variant Alzheimer’s disease) [23], or non-Alzheimer’s disease neurodegenerative disorders (*n* = 91) including frontotemporal dementia (*n* = 20), dementia with Lewy bodies (*n* = 24), Parkinson’s disease dementia (*n* = 19), progressive supranuclear palsy (*n* = 13), corticobasal syndrome (*n* = 10), vascular dementia (*n* = 3) and dementia not otherwise specified (*n* = 2). Amyloid-β status (negative/positive) was defined using either CSF (*n* = 82) or PET (*n* = 1,487), unless missing (*n* = 76).

### Tau-PET acquisition

All participants underwent Tau-PET using the [^18^F]flortaucipir radiotracer. For all cohorts the target acquisition time included the 80–100 min post-injection time interval, and all Tau-PET data were locally attenuation corrected and reconstructed into 4 × 5-minute frames according to scanner-specific protocols. Details on Tau-PET acquisition procedures for ADC, Eli Lilly and BioFINDER-1 are described previously [1, 24, 25], and a description of PET acquisition in ADNI can be found at http://adni.loni.usc.edu/methods/pet-analysis-method/pet-analysis/.

### Tau-PET visual read and TAU-SPEX

**Fig. 1:**
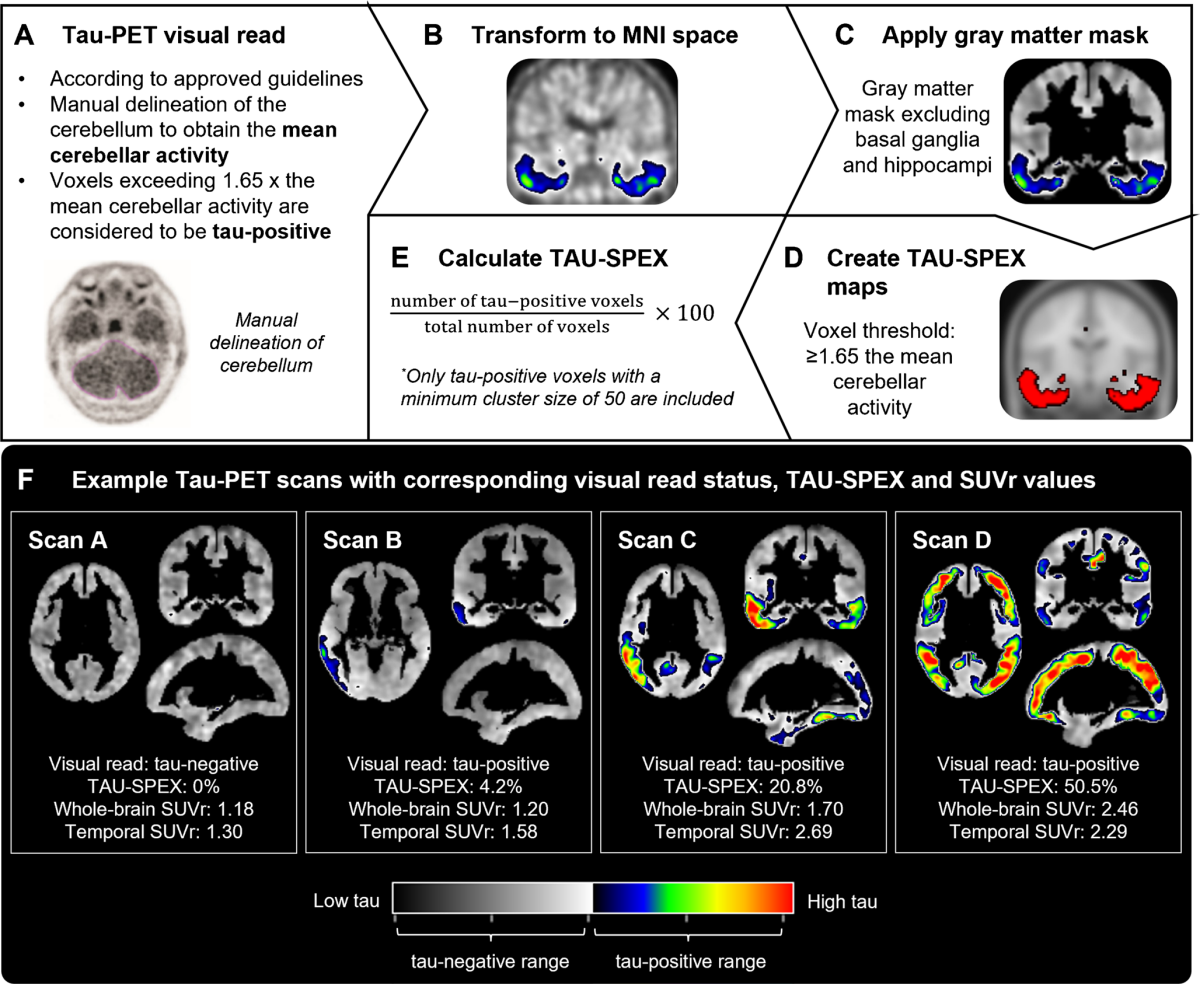
TAU-SPEX methodology. In **A**, the FDA-approved guidelines for [^18^F]flortaucipir visual read are summarized. Tau-PET images were visually read in native space independently of TAU-SPEX or SUVr. In **B**-**E**, our methodology for quantifying TAU-SPEX is shown. TAU-SPEX was developed to closely align with the existing Tau-PET visual read framework, specifically, by using a similar cerebellar scaling and threshold approach as used for visual read. In **F**, four example scans with corresponding visual read status, TAU-SPEX and SUVr values are shown. These example scans highlight the large variance present among visually tau-positive scans (Scan B, C and D) that is well-captured using TAU-SPEX. Moreover, the example scans in A and B highlight that whole-brain Tau-PET uptake is better captured using TAU-SPEX than SUVr

We aimed to develop a voxel-wise Tau-PET spatial extent metric (“TAU-SPEX”) that aligns with the existing [^18^F[flortaucipir visual read framework. Figure 1 summarizes our methodology for visual read and TAU-SPEX quantification.

First, we visually read all Tau-PET images according to guidelines approved by the FDA and EMA [11]. Tau-PET images were visually assessed without taking into account TAU-SPEX or SUVr values. Visual read was performed on 80–100 min non-intensity-normalized images (i.e. co-registered, averaged frames) for scans from the ADNI and ADC cohorts, and on 80–100 min SUVr images processed at Lund University for scans from the Eli Lilly and BioFINDER-1 cohorts. Briefly, for all scans, a region-of-interest was manually delineated around the cerebellum to obtain the average tracer activity in the cerebellum. Voxels with activity of > 65% above the cerebellar average were defined as having increased activity relative to background activity. Increased activity in posterolateral temporal, occipital, or parietal/precuneus region(s) in either hemisphere, with or without frontal involvement, resulted in a positive visual read. The absence of increased activity or increased activity isolated to medial temporal, anterolateral temporal, and/or frontal regions resulted in a negative visual read. Patterns of isolated or small non-confluent foci of increased activity were not classified as tau-positive.

Tau-PET scans from ADC were visually read by two trained nuclear medicine physicians and a consensus read was obtained in case of between-reader disagreement, as described previously [26]. Due to the large number of scans included in the current study and the time-consuming process, scans from ADNI, Eli Lilly and BioFINDER-1 were visually read by one trained reader (E.C.). E.C. also read 30 randomly selected scans from the ADC cohort to test the inter-rater agreement between reads from E.C. and the consensus read of the two readers from the ADC cohort. This resulted in a kappa of 0.92 indicating excellent agreement. In ADNI, due to significant heterogeneity in image quality, scans that were deemed difficult to interpret according to E.C. were re-examined by E.V.D.G., and a consensus read was obtained in case of between-reader disagreement.

After the visual read process, we calculated TAU-SPEX. First, we spatially normalized the PET images to Montreal Neurological Institute (MNI) space with 1.5 mm isometric voxels using Statistical Parametric Mapping (SPM) version 12 software (Wellcome Trust Centre for Neuroimaging, University College London, UK) by using the transformation matrixes derived from warping co-registered T1-weighted MRI scans to MNI space. A gray matter mask was applied in which the basal ganglia and hippocampi were excluded due to potential off-target binding in these regions [14, 27]. We then created individualized TAU-SPEX maps in which voxels were thresholded and binarized with only voxels being retained when the tau-threshold was reached. For the threshold, we used the same threshold as used within the visual read framework, i.e. each voxel was thresholded at > 65% above the manually delineated cerebellar average [11]. TAU-SPEX was calculated as the percentage of voxels exceeding the tau-threshold relative to the total number of voxels in the gray matter mask. To minimize signal resulting from noise in the TAU-SPEX metric, we only counted voxels exceeding the tau-threshold when they occurred within clusters of at least 50 voxels. We performed several sensitivity analyses, including different (i) voxel-level tau-thresholds (i.e. >65% above the cerebellar average vs. > 50% and > 75% above the cerebellar average), (ii) approaches to delineate the reference region (i.e. a manually drawn region around the cerebellum vs. an automated procedure) and (iii) type of images to calculate TAU-SPEX (i.e. non-intensity-normalized images vs. SUVr images).

### Tau-PET SUVr

As Tau-PET is typically quantified using SUVr, we additionally computed Tau-PET SUVr in both a spatially unconstrained whole-brain meta-ROI (similarly to TAU-SPEX) and temporal meta-ROI (a commonly used ROI for Tau-PET) for comparative purposes. Tau-PET scans from ADC, Eli Lilly and BioFINDER-1 were processed into SUVr images at Lund University according to previously reported procedures [1]. For ADNI, we downloaded regional Tau-PET SUVr data from the ADNI database, processed by UC Berkeley using highly similar methods. For all scans, the inferior cerebellar gray matter was used as the reference region, and regional SUVr values were extracted using the Desikan-Killiany atlas. Tau-PET SUVr in the temporal meta-ROI was calculated as the volume-weighted average of the entorhinal cortex, amygdala, parahippocampal gyrus, fusiform gyrus, and inferior and middle temporal gyrus [19]. Tau-PET SUVr in the whole-brain ROI was calculated as the volume-weighted average of all cortical regions and the subcortical amygdala region (i.e. the amygdala, banks of the superior temporal sulcus, caudal anterior cingulate, caudal middle frontal, cuneus, entorhinal, frontal pole, fusiform, inferior parietal, inferior temporal, insula, isthumus cingulate, lateral occipital, lateral orbitofrontal, middle temporal, paracentral, parahippocampal, pars operculars, pars orbitalis, pars triangularis, pericalcarine, postcentral, posterior cingulate, precentral, precuneus, rostral anterior cingulate, rostral middle forntal, superior frontal, superior parietal, superior temporal, supramaginal gyrus, temporal pole, transverse temporal gyrus). Thus, we only excluded the hippocampi and basal ganglia regions, resulting in highly similar target regions for both whole-brain SUVr and TAU-SPEX.

### Cognitive outcome measure

Baseline Mini-Mental State Examination (MMSE, the only cognitive measure acquired across all cohorts) was available for the majority of participants (1,622/1,645 [98.6%]). For longitudinal analyses, we collected MMSE scores measured up to 12 months prior to Tau-PET, as well as all MMSE scores measured after Tau-PET. Longitudinal MMSE scores were only collected for visually tau-positive individuals with MCI or Alzheimer’s disease dementia, as we aimed to investigate whether TAU-SPEX may complement Tau-PET visual read for prognostic purposes. Among *N* = 501 tau-positive participants with MCI or Alzheimer’s disease dementia and complete data on all covariates of interest (i.e. age, sex and education), we included a total of 1606 longitudinal MMSE scores, with a median of 3 per participant (range: 1–9, IQR: 2) and an average follow-up time of 1.8 ± 1.4 years.

### Neuropathology

A subset of participants (*n* = 18) had undergone Tau-PET during life and had undergone an autopsy including Braak NFT staging. The subset included *n* = 9 participants from ADC (*n* = 9 dementia at time of Tau-PET) and *n* = 9 from ADNI (*n* = 2 cognitively unimpaired, *n* = 1 MCI, and *n* = 6 dementia at time of Tau-PET). The median time between in-vivo Tau-PET imaging and postmortem Braak NFT staging was 2.0 years (range: 0–6 years, IQR: 1.75 years). We assessed both single Braak NFT stages (ranging from Braak stage I to VI) as well combined Braak NFT stages (Braak stage I-II; Braak stage III-IV and Braak stage V-VI) [28]. Given that suprathreshold Tau-PET retention has been shown to correspond to the presence of NFT pathology in Braak V-VI [11, 29, 30], we assessed the ability of TAU-SPEX and SUVr for identifying NFT pathology in Braak stages V-VI.

### Statistical analyses

All analyses were performed in R version 4.4.1. A *p*-value < 0.05 was considered statistically significant. Analyses were primarily performed across all cohorts combined, and supplementary analyses were performed stratified for each cohort.

We examined the relationship between TAU-SPEX and SUVr using linear, quadratic, and cubic models, and determined which model best fitted this relationship using the Akaike Information Criterion (AIC). We used Levene’s tests for equality of variances to test whether TAU-SPEX shows less variance in (presumably) noise-related signal compared to SUVr, while preserving or even increasing variance in “on-target” tau-signal. We determined the Area Under the Curve (AUC) of both TAU-SPEX and SUVr for discriminating visual read tau-negative from tau-positive participants and compared these AUCs using DeLong’s test. Next, we defined the optimal threshold of TAU-SPEX and SUVr for distinguishing between visual read tau-negative and tau-positive participants using the Youden’s index, and we determined the resulting accuracy, sensitivity, specificity, positive predictive value (PPV) and negative predictive value (NPV). We then assessed the agreement between Tau-PET visual read and TAU-SPEX or SUVr using Cohen’s kappa. In the subset of participants that had both ante-mortem Tau-PET and post-mortem Braak staging available, we determined the sensitivity and specificity of TAU-SPEX and SUVr for identifying NFT pathology in Braak Stages V-VI. Finally, among visually tau-positive participants with MCI or Alzheimer’s disease dementia, we assessed the association between TAU-SPEX and SUVr with concurrent performance on the MMSE and longitudinal performance on the MMSE. We performed linear mixed-effects models with random time slopes, random intercepts for subject, and random intercepts for cohort, using the lme4 package. These linear mixed-effects models were covaried for age, sex and education, and we included Tau-PET (TAU-SPEX or SUVr), time and an interaction between Tau-PET*time as fixed effects.

## Results

### Participants

A total of 1,645 participants were included, among which 1032 visually read as Tau-PET-negative (*n* = 628 cognitively unimpaired, *n* = 259 MCI, and *n* = 145 dementia) and 613 visually read as Tau-PET-positive (*n* = 60 cognitively unimpaired, *n* = 154 MCI, and *n* = 399 dementia). Participant characteristics stratified by visual read tau-status are shown in Table 1. Supplementary Table 2 shows participants characteristics for each cohort separately. A positive Tau-PET visual read was observed in 8.7% (60/688) of cognitively unimpaired individuals (23.4% [50/214] in amyloid-positive cognitively unimpaired individuals), 37.3% (154/413) of individuals with MCI (66.7% [148/222] in amyloid-positive MCI), and 73.4% (399/544) of individuals with dementia (85.8% [369/430] in amyloid-positive dementia). As expected, the visually tau-positive group consisted predominantly of individuals that were also amyloid-positive (95.5%). Four example participants including their Tau-PET visual read status, TAU-SPEX and SUVr values are shown in Fig. 1.

**Table 1:** Demographics

	All Participants	Visual Read Tau-PET-negative	Visual Read Tau-PET-positive
N	1645	1032	613
Age, y	71.9 ± 8.4	71.9 ± 8.3	72.1 ± 8.4
Sex, n female (%)	828 (50.3)	512 (49.6)	316 (51.5)
Education, y	13.6 ± 4.6	13.9 ± 4.8	13.1 ± 4.3
*APOE-*ε4 carrier, n (%)	729 (46.2)	326 (33.0)	403 (68.4)
Aβ-positive, n (%)	866 (55.2)	299 (30.7)	567 (95.5)
MMSE	25.5 ± 6.0	27.9 ± 3.2	21.4 ± 7.3
Syndrome diagnosis			
CU, n (%)	688 (41.8)	628 (60.9)	60 (9.8)
MCI, n (%)	413 (25.1)	259 (25.1)	154 (25.1)
Dementia, n (%)	544 (33.1)	145 (14.1)	399 (65.1)

Shown are mean ± standard deviation unless specified otherwise. Dementia refers to all-cause dementia. Education was missing for *n* = 69, MMSE was missing for *n* = 23; APOE-ε4 carriership for *n* = 67; and Aβ-status for *n* = 76. Aβ = amyloid-beta; CU = Cognitively Unimpaired; MCI = Mild Cognitive Impairment; MMSE = Mini-Mental State Examination

### TAU-SPEX

**Fig. 2:**
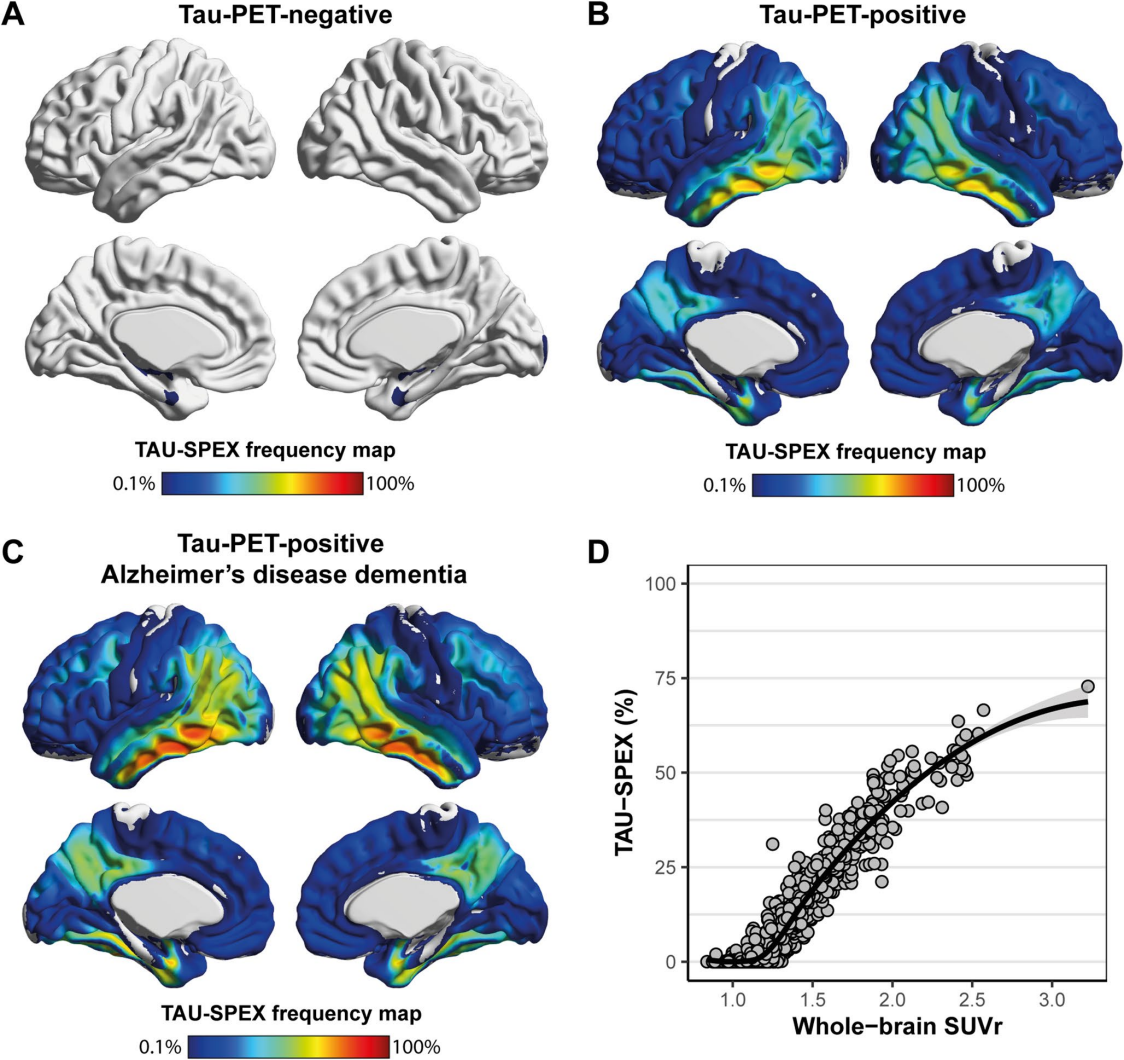
Voxel-wise TAU-SPEX frequency maps and association between TAU-SPEX and SUVr. Shown in **A**, **B**, and **C** are the TAU-SPEX voxel-wise frequency maps for visually tau-negative participants, visually tau-positive participants, and visually tau-positive participants with Alzheimer’s disease dementia respectively. These maps reflect, for each voxel, the percentage of participants whose voxel intensity exceeded the threshold. In **D**, the relationship between TAU-SPEX and whole-brain SUVr is shown. Voxel-wise maps were created using BrainNet with the “Jet” colorscale

Figure 2A-C shows the TAU-SPEX voxel-wise frequency maps for visually tau-negative participants, visually tau-positive participants, and visually tau-positive participants with Alzheimer’s disease dementia. These maps reflect, for each voxel, the percentage of participants whose voxel intensity exceeded the tau-threshold. Among tau-positive participants with Alzheimer’s disease dementia, > 75% of participants showed suprathreshold tau-tracer binding in lateral temporal voxels. Overall, the TAU-SPEX patterns showed typical patterns observed in Alzheimer’s disease, with prominent temporoparietal involvement and very limited involvement of the sensory-motor cortex.

The relationship between TAU-SPEX and SUVr was best described by a cubic polynomial curve (adjusted R^2^ cubic model: 0.921; quadratic model: 0.897; linear model: 0.895). (Fig. 2D). The initial phase shows relatively stable TAU-SPEX values (around ~ 0%) while SUVr varies. This is followed by a steep and linear increase in both TAU-SPEX and SUVr, and finally, a plateauing phase where further increases in SUVr minimally affect TAU-SPEX.

### TAU-SPEX and SUVr in visual read tau-positive and tau-negative individuals

**Fig. 3:**
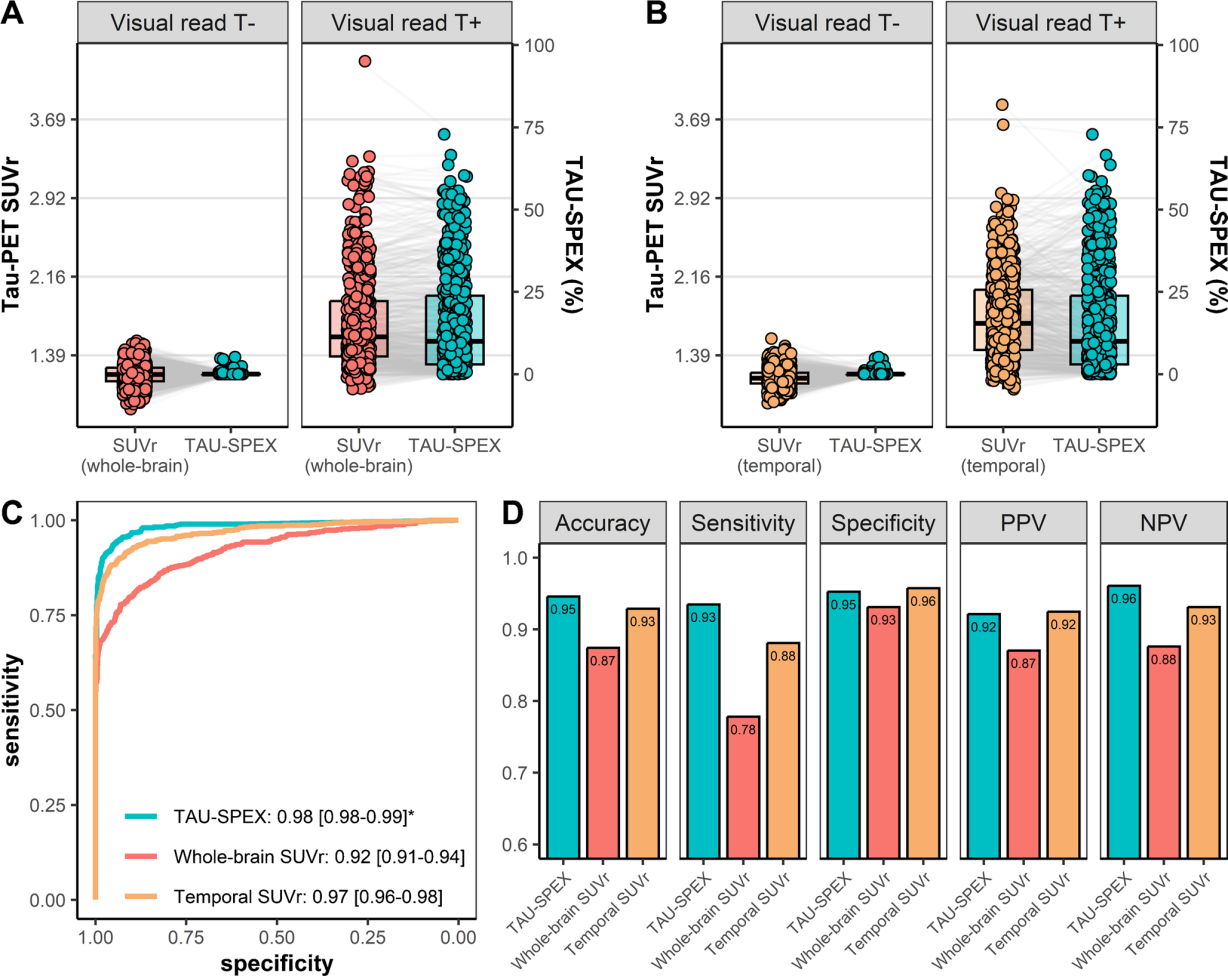
TAU-SPEX and SUVr in relation to Tau-PET visual read. Shown in **A** and **B** are TAU-SPEX and SUVr values (double y-axes) in visual read tau-negative (T-) and visual read tau-positive (T+) participants. In **A**, whole-brain SUVr is shown, and in **B** temporal meta-ROI SUVr is shown. To enable plotting TAU-SPEX and SUVr on the same axis, we z-transformed each Tau-PET metric in the total group, however, for interpretation purposes, we display raw y-axis scales instead of z-transformed y-axis scales. The scale for SUVr is shown on the left, and the scale for TAU-SPEX is shown on the right. We linked data-points from the same participants using grey lines in order to visualize the reduced variance in TAU-SPEX within visual read tau-negative participants, and the maintained (or increased) variance in TAU-SPEX within visual read tau-positive participants. In **C**, the Receiver Operating Characteristic (ROC) curves for Tau-PET visual read against TAU-SPEX, whole-brain SUVr and temporal meta-ROI SUVr are shown. In **D**, the accuracy, sensitivity, specificity, positive predictive value (PPV) and negative predictive value (NPV) for identifying a positive Tau-PET visual read are shown. *The AUC of TAU-SPEX was significantly higher than the AUC of whole-brain SUVr (*p*<0.001) and the AUC of temporal SUVr (*p*<0.001)

Among participants with a positive Tau-PET visual read, TAU-SPEX varied from 0.0 to 72.8% (median: 10.0%, interquartile range [IQR]: 20.8%), highlighting the large heterogeneity in the size of the visually tau-positive area among tau-positive individuals (Fig. 3A, B). Since the threshold used to quantify TAU-SPEX is identical to the threshold used for visual read, we performed a quality control visual inspection in scans that were visually tau-positive but had a TAU-SPEX value of 0.0% (observed in 6/613 [0.98%] of scans). All six scans showed supra-threshold tracer retention in clusters of less than 50 voxels, which were not counted in our TAU-SPEX metric as we aimed to minimize signal potentially resulting from noise in our TAU-SPEX metric. Among tau-positive participants, Tau-PET SUVr ranged from 0.98 to 3.22 SUVr (median: 1.33, IQR: 0.38 SUVr) in the whole-brain ROI, and from 1.06 to 3.83 SUVr (median: 1.70, IQR: 0.59 SUVr) in the temporal meta-ROI.

Among participants with a negative visual read, TAU-SPEX ranged from 0.0 to 5.2% (median: 0.0%, IQR: 0.03%). We performed a quality control visual inspection of scans that were visually tau-negative but had TAU-SPEX values exceeding 1.0% (observed in 10/1032 [0.97%] of scans). These scans tended to show supra-threshold tracer retention in brain areas vulnerable to off-target binding, such as the meninges/skull resulting in spill-in binding into adjacent neocortical voxels (Supplementary Fig. 1). Among tau-negative participants, Tau-PET SUVr ranged from 0.84 to 1.31 SUVr (median: 1.05, IQR: 0.09 SUVr) in the whole-brain ROI, and from 0.92 to 1.55 SUVr (median: 1.17, IQR: 0.11 SUVr) in the temporal meta-ROI (Fig. 3A, B).

One potential advantage of TAU-SPEX over SUVr is that TAU-SPEX may be less affected by subthreshold (presumably) noise-related variation while preserving (or even increasing) “on-target” tau-related variation in Tau-PET signal. To test this, we first z-transformed TAU-SPEX and SUVr within the total cohort to enable assessing variances within subgroups on the same scale. Among visually tau-negative participants (in whom low levels of tau proteinopathy are expected), there was significantly less variance in TAU-SPEX (variance: 0.0007) than in whole-brain SUVr (variance: 0.07) or temporal SUVr (variance: 0.05) (for both, Levene’s Test for comparing variances: *p* < 0.001) (Fig. 3A, B). In contrast, among visually tau-positive participants, there was significantly more variance in TAU-SPEX (variance: 1.66) than in whole-brain SUVr (variance: 1.48) and in temporal SUVr (variance: 1.12, both Levene’s Tests: *p* < 0.05). Similarly, among amyloid-negative participants, there was significantly less variance in TAU-SPEX (variance: 0.08) than in whole-brain SUVr (variance: 0.15) or temporal SUVr (variance: 0.13) (both *p* < 0.001). Among amyloid-positive participants, the variances of TAU-SPEX (variance: 1.48) and whole-brain SUVr (variance: 1.37) and temporal SUVr (variance: 1.23) were comparable (both *p* > 0.05). These results were highly consistent across all cohorts (Supplementary Fig. 2, Supplementary Table 3).

To differentiate between visual read tau-positive participants and visual read tau-negative participants, TAU-SPEX showed a significantly higher AUC (0.98 [95% CI: 0.98–0.99]) than whole-brain SUVr (0.92 [95% CI: 0.91–0.94], DeLong’s Test for comparing AUCs: *p* < 0.001) and temporal SUVr (0.97 [95% CI: 0.96–0.98], *p* < 0.001) (Fig. 3C). For TAU-SPEX, the optimal Youden-based threshold for differentiating between visually tau-positive and tau-negative individuals was 0.27%. For SUVr, the optimal Youden-based was 1.19 SUVr in the whole-brain ROI, and 1.32 in the temporal meta-ROI. The resulting accuracy, sensitivity, specificity, NPV and PPV for identifying visually tau-positive individuals are shown in Fig. 3D/Table 2, with TAU-SPEX showing strong diagnostic performance across all measures (> 0.90). TAU-SPEX showed the highest agreement with Tau-PET visual read (κ = 0.89, 94.6% concordance), followed by temporal meta-ROI SUVr (κ = 0.85, 92.9% concordance) and whole-brain SUVr (κ = 0.73, 87.4% concordance).

**Table 2:** Diagnostic performance of TAU-SPEX vs. SUVr for identifying visual read tau-positive individuals

	TAU-SPEX	SUVr (whole-brain)	SUVr (temporal meta-ROI)
Accuracy	0.95	0.87	0.93
Sensitivity	0.93	0.78	0.88
Specificity	0.95	0.93	0.96
PPV	0.92	0.87	0.92
NPV	0.96	0.88	0.93

We first obtained the optimal Youden-based threshold for TAU-SPEX and SUVr for differentiating visual read status. We then applied these thresholds to obtain the accuracy, sensitivity, specificity, positive predictive value (PPV) and negative predictive value (NPV) of TAU-SPEX and SUVr for identifying a positive visual read

### TAU-SPEX, SUVr and visual read associations with NFT Braak stage V-VI pathology at autopsy

**Fig. 4:**
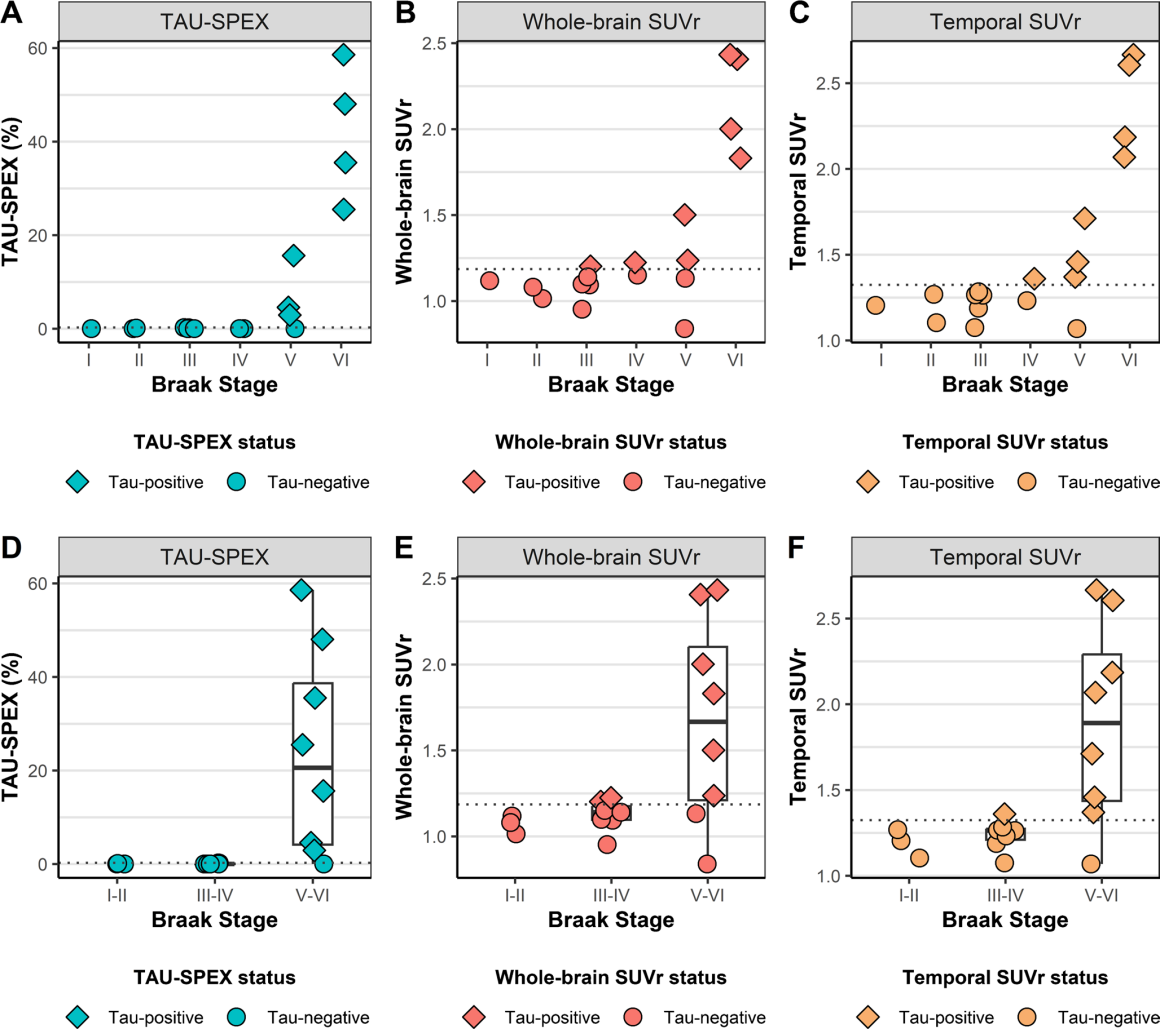
Comparing antemortem TAU-SPEX and SUVr to postmortem neurofibrillary tau tangle staging. Shown on the y-axis is ante-mortem TAU-SPEX (**A**, **D**), whole-brain SUVr (**B**, **E**) or temporal meta-ROI SUVr (**C**, **F**), and on the x-axis post-mortem NFT Braak Stage shown as single stages (**A**, **B**, and **C**) and combined stages (**D**, **E**, and **F**). Datapoints are colored on TAU-SPEX status, whole-brain SUVr status or temporal SUVr status as defined using the Youden-derived thresholds in our previous analysis (see Fig. 3). The dotted lines reflect each Youden-derived threshold. A box plot was only included when at least 8 datapoints were available. The one case with Braak-V NFT pathology that fell below the threshold of both TAU-SPEX, whole-brain SUVr and temporal SUVr had a time interval of 2.46 years between PET and death. The plot includes *n* = 1 Braak-I, *n* = 2 Braak-II, *n* = 5 Braak-III, *n* = 2 Braak-IV, *n* = 4 Braak-V and *n* = 4 Braak-VI cases. Some datapoints may overlap due to having similar or identical values

To further test the performance of TAU-SPEX to detect actual tau-related signal, we examined its sensitivity and specificity for detecting NFT pathology in advanced Braak stages (i.e. Braak stage V-VI) and compared it against both SUVr and visual read. In Fig. 4, we show the antemortem TAU-SPEX, whole-brain SUVr, and temporal meta-ROI SUVr values against postmortem NFT Braak stages in *n* = 18 participants who had undergone Tau-PET during life and had undergone an autopsy. TAU-SPEX had a sensitivity of 87.5% and a specificity of 100.0% for detecting Braak V-VI neuropathology. Whole-brain SUVr showed both a lower sensitivity (75.0%) and specificity (80.0%), and temporal meta-ROI SUVr showed a comparable sensitivity (87.5%) but a lower specificity (90.0%). Tau-PET visual read also showed a comparable sensitivity (87.5%) but a lower specificity (80.0%).

### TAU-SPEX and SUVr associations with cognitive decline in visually tau-positive individuals

Finally, we aimed to investigate whether TAU-SPEX can complement Tau-PET visual read by providing prognostic information within visually tau-positive individuals with MCI or Alzheimer’s disease dementia. Among visually tau-positive participants with MCI or Alzheimer’s disease dementia, higher TAU-SPEX was associated with lower cross-sectional MMSE scores (standardized β=−0.36 [−0.30, −0.43], *p* < 0.001) and steeper rates of MMSE decline (β=−0.19 [−0.15, −0.22], *p* < 0.001). Both whole-brain and temporal meta-ROI SUVr were also associated with lower cross-sectional MMSE scores (whole-brain: β=−0.32 [−0.25, −0.39], *p* < 0.001; temporal: β=−0.30 [−0.24, −0.37], *p* < 0.001) and steeper rates of MMSE decline (whole-brain: β=−0.15 [−0.11, −0.19], *p* < 0.001; temporal: β=−0.17 [−0.13, −0.21], *p* < 0.001) (Supplementary Fig. 3). We found better model fits for the model including TAU-SPEX as predictor compared to the model including whole-brain SUVr as predictor (ΔAICc TAU-SPEX: 3024.3– SUVr: 3067.6 = −43.3) as well as compared to the model including temporal SUVr as predictor (ΔAICc TAU-SPEX: 3024.3– SUVr: 3053.4 = 29.1). Comparable results were found for each cohort separately, for which the results are shown in Supplementary Table 4.

### Sensitivity analyses

We performed several sensitivity analyses with regards to our TAU-SPEX methodology. To align with the visual read framework, TAU-SPEX is calculated by thresholding voxels at > 65% above the manually delineated cerebellar average. In scans from ADC, we compared TAU-SPEX calculated using the manually delineated cerebellar average activity to TAU-SPEX calculated using an automated, atlas-delineated (Desikan-Killiany) cerebellar average, as the latter approach might minimize user influence and enhance clinical applicability. We observed a strong correlation (*r* = 0.990) indicating comparable TAU-SPEX values when using a manual versus automated delineation of the cerebellum. Furthermore, in scans from ADC, we correlated TAU-SPEX calculated from non-intensity-normalized images (as used for ADNI and ADC) to TAU-SPEX calculated from SUVr images (as used for Eli Lilly and BioFINDER-1) to establish whether differences in pre-processing steps may affect TAU-SPEX. We observed a strong correlation (*r* = 0.998), again indicating a minimal influence of minor methodological deviations on TAU-SPEX values. Finally, we assessed whether different voxel-level thresholds for calculating TAU-SPEX (> 65% vs. > 50% and > 75% the cerebellar average) affected the sensitivity and specificity of TAU-SPEX for identifying Braak-V/VI pathology at autopsy (the gold standard for assessing the presence of neurofibrillary tangle pathology). In the subset with Braak staging available (*n* = 18), TAU-SPEX showed a sensitivity of 87.5% for identifying Braak-V/VI pathology for all three threshold approaches (> 50%, > 65% and > 75% the cerebellar average). However, whereas a threshold approach of > 65% and > 75% the cerebellar average both resulted in a specificity of 100.0%, a threshold approach of > 50% the cerebellar average resulted in a lower specificity of 60.0%.

## Discussion

In this study, we aimed to develop a spatially unconstrained voxel-wise Tau-PET spatial extent metric (“TAU-SPEX”) that aligns with the Tau-PET visual read framework and captures interindividual variability in the size of the visually tau-positive area. We showed that TAU-SPEX had high accuracy, sensitivity, specificity, PPV and NPV (all > 0.90) for distinguishing visually tau-negative from tau-positive individuals. Moreover, TAU-SPEX had high sensitivity and specificity for distinguishing between individuals with and without Braak-V/VI NFT pathology at autopsy. This underscores the accuracy of TAU-SPEX in identifying neocortical tau pathology. Finally, among visually tau-positive individuals with MCI and Alzheimer’s disease dementia, TAU-SPEX was associated to both concurrent and longitudinal cognitive performance. Across analyses, TAU-SPEX generally outperformed SUVr. Our findings indicate that TAU-SPEX is an accurate measure of whole-brain tau proteinopathy, and may potentially be a suitable metric to be used in combination with visual read in clinical settings, for example by supporting the visual read process for diagnostic applications or by complementing the visual read results for prognostic purposes.

Given that visually tau-positive PET scans can substantially vary in the size of the (visually) tau-positive area, we aimed to develop a spatial extent metric that would capture this variability. Therefore, we developed a voxel-wise spatial extent metric that aligns with the Tau-PET visual read framework, hence applying a different methodology compared to previous spatial extent approaches [15–18]. First, whereas some previous studies quantified spatial extent at an atlas-based ROI-level, we quantified TAU-SPEX at the voxel-level thereby amplifying its advantage of being spatially unconstrained. Second, whereas some previous studies quantified spatial extent using relatively low thresholds, we applied conservative voxel-level tau-thresholds thereby preserving clinically meaningful signal and minimizing noise. The voxel-level tau-threshold used to binarize each voxel was identical to the threshold used to visual read each Tau-PET image, i.e. >65% above the cerebellar average. Of note, while it has not been demonstrated that voxels > 65% above the cerebellar average reflect locally elevated NFT pathology, studies investigating the Tau-PET visual read method showed that visually tau-positive scans corresponded strongly to the presence of NFT Braak-V/VI neuropathology [11], and were associated with the presence of amyloid-pathology, disease stage, and clinical progression [26, 31]. In our TAU-SPEX methodology, we additionally employed a minimum cluster size of 50 tau-positive voxels to be included in the TAU-SPEX metric. This was done to align with the visual read method (since isolated or small non-confluent foci of increased activity do not contribute to a positive scan), and to minimize signal potentially resulting from noise in our TAU-SPEX metric. Due to the relatively high threshold and the minimum cluster size, TAU-SPEX is minimally influenced by noise-related signal and foci, thereby more accurately capturing actual neocortical tau proteinopathy. This is corroborated by our finding that, compared to SUVr, TAU-SPEX showed significantly less variance in individuals who are expected to have low levels of tau (e.g. tau-negative and/or amyloid-negative individuals), while, importantly, TAU-SPEX increased or retained comparable variance compared to SUVr in individuals expected to have tau pathology. The hypothesized increased sensitivity and specificity of TAU-SPEX for tau tangle pathology is further corroborated by our comparison analyses against tau tangle pathology at autopsy, indicating highest sensitivity and specificity to NFT Braak-V/VI pathology. Moreover, when applying a lower voxel-level threshold for calculating TAU-SPEX (> 50% instead of > 65% the cerebellar average), a similar sensitivity but a substantially lower specificity for identifying Braak-V/VI NFT pathology was observed, supporting the use of a relatively high threshold for calculating TAU-SPEX. Together, these findings show that TAU-SPEX is an accurate and sensitive metric for identifying the presence of neocortical tau pathology.

TAU-SPEX generally outperformed SUVr in the analyses performed in this study. Previous studies investigating spatial extent metrics similarly showed either superior or non-inferior performance of spatial extent compared to SUVr in associations with cognition and amyloid-β pathology [15–17]. Tau-PET SUVr in the temporal meta-ROI has shown strong associations with cognitive stage, amyloid-β pathology, cognitive decline and clinical progression in both early and later stages of the disease [5, 13, 19]. However, in individuals with atypical Tau-PET uptake patterns, or individuals with advanced disease in which tau has spread beyond the temporal lobe, the temporal meta-ROI might inadequately capture all relevant tau burden. Our findings show that when a spatially unconstrained whole-brain region is of interest, TAU-SPEX is a more suitable metric than whole-brain SUVr. This might especially be relevant in associations with cognition, as tau outside of the temporal lobe strongly impact clinical trajectories [9]. Among individuals labeled as visual read tau-positive, we observed moderate associations between TAU-SPEX and concurrent functioning on the MMSE as well as longitudinal performance on the MMSE. This suggests that once a tau-positive diagnosis is made based on the dichotomous visual read result, quantitative TAU-SPEX values may add complementary information valuable for prognostic purposes.

There has been increasing interest in the field to combine Tau-PET visual reads with quantitative Tau-PET metrics [12]. Moreover, a recent study demonstrated that the individual workload of nuclear medicine physicians increased significantly in recent years due to a larger demand [32]. With the recent approval of several therapeutic agents for Alzheimer’s disease [33, 34], the demand for nuclear medicine procedures in the dementia field might further increase in the coming years. Disclosing a quantitative Tau-PET metric to nuclear medicine physicians during the visual read process may improve the accuracy of the visual read and could result in increased reader confidence. Moreover, a quantitative metric may be used in future to filter out clearly positive and negative scans, which may reduce the number of visual reads needed to perform in trials and potentially fasten the visual read process in clinical practice. Our findings show that TAU-SPEX may be a suitable metric for this purpose. TAU-SPEX demonstrated strong performance in discriminating visual read tau-negative from visual read tau-positive individuals (AUC: 0.97). Moreover, TAU-SPEX showed high accuracy, sensitivity, specificity, PPV and NPV values (all > 0.90) for identifying visually tau-positive individuals, which was also highly consistent across cohorts. An additional advantage of TAU-SPEX is that TAU-SPEX is easily interpretable and tangible for patients, caregivers and clinicians. The well-defined starting point (0%) and ceiling (100%) enable easy understanding and communication of tau pathological progression at the individual level. Disclosure of TAU-SPEX values to nuclear medicine physicians may therefore serve as a supportive instrument to the Tau-PET visual read process, similar to how SUVr or Centiloid values can support the visual assessment of Amyloid-PET [35]. A prospective study in the context of the Dutch TAP-TAU study in which TAU-SPEX values will be disclosed during the visual read process will be valuable to fully understand the value of combining visual reads with quantitative metrics in clinic [36]. 

TAU-SPEX is specifically developed for [^18^F]flortaucipir PET images, while other tau-binding radiotracers (such as [^18^F]MK6240, [^18^F]RO948 and [^18^F]PI2620) are also commonly used. As each tracer has different tracer properties and off-target binding profiles, future studies should examine whether our TAU-SPEX methodology (using the voxel-level threshold of > 65% the cerebellar average) can also be applied to other tau-binding radiotracers. In this regard, if this threshold is also applicable to other tau-binding radiotracers, TAU-SPEX may be of interest in the context of harmonizing and pooling Tau-PET data from different tau-binding radiotracers and different cohorts, for which efforts are currently on-going [37–39]. Using TAU-SPEX as a universal Tau-PET scale might only require harmonizing the voxel-level threshold instead of harmonizing the entire range of SUVr values. Of note, in our study, the performance of TAU-SPEX was consistent across all four cohorts included. Although TAU-SPEX has the advantage of accurately capturing tau in a spatially unconstrained manner, a potential disadvantage is that it fully disregards the location of the voxels exceeding the tau-threshold, while the location of “tau-positive” voxels likely has meaningful information– for example, voxels exceeding the threshold in the frontal lobe may have different clinical consequences than voxels exceeding the threshold in the temporal lobe. Moreover, TAU-SPEX does not take information on tracer intensity into account (both sub-threshold and supra-threshold intensity). It may be of interest to combine TAU-SPEX with SUVr metrics, or to investigate a “weighted” TAU-SPEX approach in which voxels can be given more weight depending on their association with specific cognitive functions or specific Alzheimer’s disease phenotypes. Furthermore, given that TAU-SPEX is a spatially unconstrained metric that is not limited to an a-priori defined ROI, understanding the value of TAU-SPEX as compared to SUVr in atypical variants of Alzheimer’s disease (who do not follow the typical tau trajectories captured by the temporal meta-ROI [40]), as well as in relation to domain-specific cognitive tests, would be of interest. Additionally, an open-access tool that automates the methodology for quantifying TAU-SPEX and generating the corresponding TAU-SPEX image (such that the location of supra-threshold voxels can be taken into account) will be important to enhance implementation and generalizability to the clinic. Finally, the longitudinal value of TAU-SPEX, for example as a disease monitoring tool or as a surrogate endpoint in trials, will be of interest to investigate further.

Strengths of this study include the use of four different cohorts, the large sample size, and the use of both quantification and visual read, thereby utilizing the full potential of Tau-PET. There are also several limitations to this work. First, the sample size for the ante-mortem Tau-PET and post-mortem Braak staging analyses was small (*n* = 18) due to limited cases with both antemortem Tau-PET and autopsy data available. TAU-SPEX may need to be quantified in studies with larger sample sizes with both antemortem Tau-PET and postmortem Braak staging available to fully understand its potential for identifying Braak V-VI neuropathology. Second, there were small methodological differences in TAU-SPEX quantification among cohorts, as TAU-SPEX in the ADC and ADNI cohorts was quantified on non-intensity normalized PET images whereas TAU-SPEX in the Eli Lilly and BioFINDER-1 cohorts was quantified on SUVr images. Third, the aim of the study was to develop a voxel-wise spatial extent metric that aligns with the [^18^F]flortaucipir visual read framework and captures interindividual variability in the size of the visually tau-positive area, and we therefore used an identical threshold for TAU-SPEX as for visual read. While this resulted in strong correspondence between TAU-SPEX and visual read (which makes TAU-SPEX a suitable supportive instrument for visual read), this may have also led to some circularity when comparing TAU-SPEX to visual read which should be taken into account when interpreting the findings. Finally, in the current study, we retrospectively included participants who underwent Tau-PET from four different cohorts and performed group-level analyses. To fully understand the diagnostic and prognostic potential of TAU-SPEX, individualized analyses, and prospective studies are preferred.

## Conclusion

TAU-SPEX–a spatially unconstrained measure reflecting the percentage of gray matter tissue affected by tau aggregates– is strongly associated with Tau-PET visual read, NFT Braak stage-V/VI pathology at autopsy, and cognitive decline. TAU-SPEX might be useful in clinical settings for example as a supportive instrument to the visual read process, or as a complementary instrument for prognostic purposes. For assessments of whole-brain tau proteinopathy, TAU-SPEX might be better suitable than whole-brain SUVr.

## Supplementary Information

Below is the link to the electronic supplementary material.

Supplementary Material 1

## Data Availability

Given that this study included four different cohorts, requests to access to individual participant data from each cohort will have to be made through the principle investigators of the respective cohorts. Generally, anonymized data used in this study can be shared by request from qualified academic investigators for the purpose of replicating procedures and results presented in the article, if data transfer is in agreement with the data protection regulation at the institution and is approved by the local Ethics Review Board.

## Abbreviations

ADC: Amsterdam Dementia Cohort
ADNI: Alzheimer’s Disease Neuroimaging Initiative
AIC: Akaike Information Criterium
AUC: Area Under the Curve
MCI: Mild Cognitive Impairment
NPV: Negative Predictive Value
PET: Positron Emission Tomography
PPV: Positive Predictive Value
SUVr: Standardized Uptake Value ratio
TAU-SPEX: Tau-PET spatial extent
