# Correction to: Quantitation of PET spatial extent as a potential adjunct to visual interpretation of [^18^F]flortaucipir imaging: TAU-SPEX

**DOI:** 10.1007/s00259-025-07434-5

**Published:** 2025-07-04

**Authors:** Emma M. Coomans, Bastiaan van Tol, Colin Groot, Ruben Smith, Sebastian Palmqvist, Erik Stomrud, Michael J. Pontecorvo, Sergey Shcherbinin, Ian Kennedy, Vikas Kotari, Wiesje M. van der Flier, Yolande A. L. Pijnenburg, Niklas Mattsson-Carlgren, Oskar Hansson, Elsmarieke van de Giessen, Rik Ossenkoppele

**Affiliations:** 1 Alzheimer Center Amsterdam, Neurology, Vrije Universiteit Amsterdam, Amsterdam UMC location VUmc, Amsterdam, The Netherlands; 2 Amsterdam Neuroscience, Neurodegeneration, Amsterdam, The Netherlands; 3 Clinical Memory Research Unit, Department of Clinical Sciences in Malmö, Lund University, Lund, Sweden; 4 Memory Clinic, Skåne University Hospital, Malmö, Sweden; 5 Eli Lilly and Company, Indianapolis, IN USA; 6 Wallenberg Center for Molecular Medicine, Lund University, Lund, Sweden; 7 Radiology & Nuclear Medicine, Vrije Universiteit Amsterdam, Amsterdam UMC location VUmc, Amsterdam, The Netherlands; 8 Amsterdam Neuroscience, Brain Imaging, Amsterdam, The Netherlands

**Correction to: European Journal of Nuclear Medicine and Molecular Imaging**

10.1007/s00259-025-07384-y

The authors regret that the legends of the figures that appeared in the original published article are incorrect.

Below are the correct and incorrect figure legends.

**Figure 1**

Correct legend: Fig. 1 TAU-SPEX methodology. In A, the FDA-approved guidelines for [18F]flortaucipir visual read are summarized. Tau-PET images were visually read in native space independently of TAU-SPEX or SUVr. In B-D, our methodology for quantifying TAU-SPEX is shown. TAU-SPEX was developed to closely align with the existing Tau-PET visual read framework, specifically, by using a similar cerebellar scaling and threshold approach as used for visual read. In F, four example scans with corresponding visual read status, TAU-SPEX and SUVr values are shown. These example scans highlight the large variance present among visually tau-positive scans (Scan B, C and D) that is well-captured using TAU-SPEX. Moreover, the example scans in A and B highlight that whole-brain Tau- PET uptake is better captured using TAU-SPEX than SUVr.

Incorrect legend: Fig. 1 TAU-SPEX methodology

**Figure 2**

Correct legend: Fig. 2 Voxel-wise TAU-SPEX frequency maps and association between TAU-SPEX and SUVr. Shown in A, B, and C are the TAU-SPEX voxel-wise frequency maps for visually tau-negative participants, visually tau-positive participants, and visually tau-positive participants with Alzheimer’s disease dementia respectively. These maps reflect, for each voxel, the percentage of participants whose voxel intensity exceeded the threshold. In D, the relationship between TAU-SPEX and whole-brain SUVr is shown. Voxel-wise maps were created using BrainNet with the “Jet” colorscale.

Incorrect legend: Fig. 2 Voxel-wise TAU-SPEX frequency maps and association between TAU-SPEX and SUVr

**Figure 3**

Correct legend: Fig. 3 TAU-SPEX and SUVr in relation to Tau-PET visual read. Shown in A and B are TAU-SPEX and SUVr values (double y-axes) in visual read tau-negative (T-) and visual read tau-positive (T+) participants. In A, whole-brain SUVr is shown, and in B temporal meta-ROI SUVr is shown. To enable plotting TAU-SPEX and SUVr on the same axis, we z-transformed each Tau-PET metric in the total group, however, for interpretation purposes, we display raw y-axis scales instead of z-transformed y-axis scales. The scale for SUVr is shown on the left, and the scale for TAU-SPEX is shown on the right. We linked data-points from the same participants using grey lines in order to visualize the reduced variance in TAU-SPEX within visual read tau-negative participants, and the maintained (or increased) variance in TAU-SPEX within visual read tau-positive participants. In C, the Receiver Operating Characteristic (ROC) curves for Tau-PET visual read against TAU-SPEX, whole-brain SUVr and temporal meta-ROI SUVr are shown. In D, the accuracy, sensitivity, specificity, positive predictive value (PPV) and negative predictive value (NPV) for identifying a positive Tau-PET visual read are shown. *The AUC of TAU-SPEX was significantly higher than the AUC of whole-brain SUVr (*p* < 0.001) and the AUC of temporal SUVr (*p* < 0.001).

Incorrect legend: Fig. 3 TAU-SPEX and SUVr in relation to Tau-PET visual read.

**Figure 4**

Correct legend: Fig. 4 Comparing antemortem TAU-SPEX and SUVr to postmortem neurofibrillary tau tangle staging. Shown on the y-axis is ante-mortem TAU-SPEX (A, D), whole-brain SUVr (B, E) or temporal meta-ROI SUVr (C, F), and on the x-axis post-mortem NFT Braak Stage shown as single stages (A, B, and C) and combined stages (D, E, and F). Datapoints are colored on TAU-SPEX status, whole-brain SUVr status or temporal SUVr status as defined using the Youden-derived thresholds in our previous analysis (see Fig. 3). The dotted lines reflect each Youden-derived threshold. A box plot was only included when at least 8 datapoints were available. The one case with Braak-V NFT pathology that fell below the threshold of both TAU-SPEX, whole-brain SUVr and temporal SUVr had a time interval of 2.46 years between PET and death. The plot includes *n* = 1 Braak-I, *n* = 2 Braak-II, *n* = 5 Braak-III, *n* = 2 Braak-IV, *n* = 4 Braak-V and *n* = 4 Braak-VI cases. Some datapoints may overlap due to having similar or identical values.

Incorrect legend: Fig. 4 Comparing antemortem TAU-SPEX and SUVr to postmortem neurofibrillary tau tangle staging

The original article has been corrected.

