# Supplementary material for: Quantitation of PET spatial extent as a potential adjunct to visual interpretation of [18F]flortaucipir imaging: TAU-SPEX
Source: Eur J Nucl Med Mol Imaging. 2025 Jun 7;52(13):5135–49. doi: 10.1007/s00259-025-07384-y (PMC12589304; doi:10.1007/s00259-025-07384-y)
Supplement: Supplementary file 1 — Supplementary Material 1 [file 259_2025_7384_MOESM1_ESM.docx]

**Supplemental Materials**

**Supplementary Table 1** Details on each cohort

| **Cohort** | **Cohort description** |
| --- | --- |
| ADC (van der Flier & Scheltens, 2018) | The Amsterdam Dementia Cohort (ADC) is a prospective cohort study including (amongst others) individuals with subjective cognitive decline, mild cognitive impairment and dementia presenting at the Alzheimer Center of the VU University Medical Center Amsterdam. All participants have been referred to the memory clinic by their general practitioner, and a neurologist or geriatrician in the case of a second opinion for evaluation of cognitive complaints. They receive standardized dementia screening at the memory clinic, including an interview with a neurologist, physical and neurological examination, neuropsychological assessment. |
| BioFINDER-1 (Ossenkoppele et al., 2018) | The Swedish BioFINDER-1 (Biomarkers Identifying Neurodegenerative Disorders Early and Reliably) study was launched in 2009 to learn more about the underlying etiologies of Alzheimer’s and Parkinson’s diseases and related disorder. More than 1600 patients with mild cognitive symptoms, dementia and parkinsonian symptoms as well as cognitively healthy elderly were enrolled in the study with longitudinal follow-up up to 10 years. The subjects underwent repeated examinations of advanced MRI (including fMRI, DTI, DKI, ASL and MPRAGE), CSF and plasma analysis, amyloid PET, detailed clinical assessments and neuropsychological examinations. A subset of study participants underwent tau PET imaging. |
| Eli Lilly (Pontecorvo et al., 2019) | The Eli Lilly cohort consisted of research participants from the A04 [NCT01565343], A05 [NCT02016560], A08 [NCT04468347], placebo arm of the Eli Lilly solanezumab Expedition-3 trial [NCT01900665], and placebo arm of the Eli Lilly LY3202626 NAVIGATE-AD trial [NCT02791191] studies. Details on in- and exclusion criteria for each specific study can be found at clinicaltrials.gov. |
| ADNI (Petersen et al., 2010) | Data used in the preparation of this article were obtained from the Alzheimer’s Disease Neuroimaging Initiative (ADNI) database (adni.loni.usc.edu). The ADNI was launched in 2003 as a public-private partnership, led by Principal Investigator Michael W. Weiner, MD. The primary goal of ADNI has been to test whether serial magnetic resonance imaging (MRI), positron emission tomography (PET), other biological markers, and clinical and neuropsychological assessment can be combined to measure the progression of mild cognitive impairment and early Alzheimer’s disease. |

**Supplementary Table 2** Participant characteristics for each cohort separately

| **ADC** | | |
| --- | --- | --- |
|  | **Visual Read Tau-PET-negative** | **Visual Read Tau-PET-positive** |
| **N** | 89 | 92 |
| **Age, y** | 67.0 ± 7.4 | 65.1 ± 7.1 |
| **Sex, n female (%)** | 32 (36.0) | 45 (48.9) |
| **Education, y** | 12.3 ± 2.8 | 12.2 ± 3.1 |
| ***APOE-*ε4 carrier, n (%)** | 32 (41.6) | 62 (70.5) |
| **Aβ-positive, n (%)** | 31 (36.0) | 91 (98.9) |
| **MMSE** | 27.4 ± 3.4 | 22.1 ± 4.6 |
| **Syndrome diagnosis** |  |  |
| **CU, n (%)** | 61 (68.5) | 6 (6.5) |
| **MCI, n (%)** | 3 (3.4) | 10 (10.9) |
| **Dementia, n (%)** | 25 (28.1) | 76 (82.6) |
| **BioFINDER-1** | | |
|  | **Visual Read Tau-PET-negative** | **Visual Read Tau-PET-positive** |
| **N** | 137 | 101 |
| **Age, y** | 71.2 ± 7.7 | 71.4 ± 7.1 |
| **Sex, n female (%)** | 63 (46.0) | 42 (41.6) |
| **Education, y** | 12.3 ± 3.7 | 12.0 ± 3.8 |
| ***APOE-*ε4 carrier, n (%)** | 55 (43.7) | 65 (67.0) |
| **Aβ-positive, n (%)** | 46 (44.2) | 83 (87.4) |
| **MMSE** | 26.5 ± 4.5 | 22.7 ± 5.1 |
| **Syndrome diagnosis** |  |  |
| **CU, n (%)** | 72 (52.6) | 6 (5.9) |
| **MCI, n (%)** | 14 (10.2) | 25 (24.8) |
| **Dementia, n (%)** | 51 (37.2) | 70 (69.3) |
| **Eli Lilly** | | |
|  | **Visual Read Tau-PET-negative** | **Visual Read Tau-PET-positive** |
| **N** | 227 | 258 |
| **Age, y** | 72.1 ± 9.1 | 72.8 ± 8.3 |
| **Sex, n female (%)** | 111 (48.9) | 143 (55.4) |
| **Education, y** | 7.8 ± 5.1 | 12.1 ± 5.1 |
| ***APOE-*ε4 carrier, n (%)** | 66 (30.7) | 167 (68.7) |
| **Aβ-positive, n (%)** | 70 (31.0) | 247 (95.7) |
| **MMSE** | 27.2 ± 4.3 | 18.1 ± 8.9 |
| **Syndrome diagnosis** |  |  |
| **CU, n (%)** | 118 (52.0) | 22 (8.5) |
| **MCI, n (%)** | 71 (31.3) | 43 (16.7) |
| **Dementia, n (%)** | 38 (16.7) | 193 (74.8) |
| **ADNI** | | |
|  | **Visual Read Tau-PET-negative** | **Visual Read Tau-PET-positive** |
| **N** | 579 | 162 |
| **Age, y** | 72.7 ± 8.0 | 75.2 ± 7.9 |
| **Sex, n female (%)** | 306 (52.8) | 86 (53.1) |
| **Education, y** | 16.5 ± 2.5 | 15.8 ± 2.3 |
| ***APOE-*ε4 carrier, n (%)** | 173 (30.3) | 109 (67.7) |
| **Aβ-positive, n (%)** | 152 (27.2) | 146 (98.0) |
| **MMSE** | 28.6 ± 1.9 | 25.2 ± 4.0 |
| **Syndrome diagnosis** |  |  |
| **CU, n (%)** | 377 (65.1) | 26 (16.0) |
| **MCI, n (%)** | 171 (29.5) | 76 (46.9) |
| **Dementia, n (%)** | 31 (5.4) | 60 (37.0) |

Shown are mean ± standard deviation unless specified otherwise. Dementia refers to all-cause dementia. For ADC, education was missing for n=6; APOE-ε4 carriership for n=16; and Aβ-status for n=3. For BioFINDER-1, education was missing for n=2; MMSE for n=3; APOE-ε4 carriership for n=15; and Aβ-status for n=39. For Eli Lilly, education was missing for n=61; MMSE for n=13; and APOE-ε4 carriership for n=27. For ADNI, MMSE was missing for n=7; APOE-ε4 carriership for n=9; and Aβ-status for n=33. Aβ = amyloid-beta; ADNI = Alzheimer’s Disease Neuroimaging Initiative; ADC = Amsterdam Dementia Cohort; CU = Cognitively Unimpaired; MCI = Mild Cognitive Impairment; MMSE = Mini-Mental State Examination.

**Supplementary Table 3** Variances of TAU-SPEX and whole-brain Tau-PET SUVr and Levene’s p-values

| **Cohort** | **Tau-status or amyloid-status** | **Variance in  TAU-SPEX** | **Variance in  whole-brain SUVr** |
| --- | --- | --- | --- |
| **ADC** | **Tau-negative** | **0.001^a^** | 0.037 |
|  | **Tau-positive** | 1.154 | 1.027 |
|  | **Amyloid-negative** | **0.001^a^** | 0.022 |
|  | **Amyloid-positive** | 1.178 | 1.111 |
| **BF1** | **Tau-negative** | **0.001^a^** | 0.040 |
|  | **Tau-positive** | 1.549 | 1.455 |
|  | **Amyloid-negative** | 0.576 | 0.714 |
|  | **Amyloid-positive** | 1.309 | 1.227 |
| **Eli Lilly** | **Tau-negative** | **0.001^a^** | 0.083 |
|  | **Tau-positive** | 1.211 | 1.102 |
|  | **Amyloid-negative** | **0.014^a^** | 0.094 |
|  | **Amyloid-positive** | 1.206 | 1.126 |
| **ADNI** | **Tau-negative** | **0.001^a^** | 0.137 |
|  | **Tau-positive** | 3.036 | 2.250 |
|  | **Amyloid-negative** | **0.006^a^** | 0.144 |
|  | **Amyloid-positive** | 2.028 | 1.719 |

Shown are the variances of TAU-SPEX and whole-brain SUVr within tau-negative, tau-positive, amyloid-negative and amyloid-positive participants in each cohort. We first z-transformed TAU-SPEX and SUVr within each cohort to enable comparing variances within groups on the same scale. Differences in variances between TAU-SPEX and SUVr were assessed using Levene’s Test for Homogeneity of Variance.

^a^ *p*<0.001; ^b^ *p*<0.05

**Supplementary Table 4** Associations of TAU-SPEX and SUVr with cross-sectional MMSE and longitudinal MMSE in visually tau-positive individuals with mild cognitive impairment or Alzheimer’s disease dementia

|  |  | **Effect on cross-sectional MMSE** | | | **Effect on longitudinal** **MMSE** | | |
| --- | --- | --- | --- | --- | --- | --- | --- |
|  |  | **TAU-SPEX** | **Whole-brain SUVr** | **Temporal SUVr** | **TAU-SPEX** | **Whole-brain SUVr** | **Temporal SUVr** |
| **ADC** | Estimate  [95% CI] | -0.47  [-0.26, -0.69] | -0.43  [-0.23, -0.64] | -0.38  [-0.19, -0.58] | -0.21  [-0.13, -0.29] | -0.20  [-0.11, -0.29] | -0.19  [-0.11, -0.27] |
|  | P-value | <0.001 | <0.001 | <0.001 | <0.001 | <0.001 | <0.001 |
|  | AIC* | 476.9 | 481.2 | 483.0 | 476.9 | 481.2 | 483.0 |
| **BF1** | Estimate  [95% CI] | -0.41  [-0.21, -0.61] | -0.39  [-0.19, -0.60] | -0.39  [-0.19, -0.58] | -0.10  [-0.03, -0.17] | -0.10  [-0.03, -0.17] | -0.12  [-0.05, -0.19] |
|  | P-value | <0.001 | <0.001 | <0.001 | 0.006 | 0.009 | 0.009 |
|  | AIC* | 556.7 | 559.7 | 554.4 | 556.7 | 559.7 | 554.4 |
| **Eli Lilly** | Estimate  [95% CI] | -0.23  [-0.14, -0.32] | -0.16  [-0.07, -0.25] | -0.19  [-0.11, -0.28] | -0.26  [-0.17, -0.35] | -0.22  [-0.13, -0.31] | -0.24  [-0.16, -0.33] |
|  | P-value | <0.001 | <0.001 | <0.001 | <0.001 | <0.001 | <0.001 |
|  | AIC* | 1407.3 | 1423.3 | 1413.6 | 1407.3 | 1423.3 | 1413.6 |
| **ADNI** | Estimate  [95% CI] | -0.38  [-0.26, -0.49] | -0.36  [-0.24, -0.48] | -0.32  [-0.21, -0.44] | -0.22  [-0.16, -0.28] | -0.20  [-0.13, -0.26] | -0.21  [-0.14, -0.27] |
|  | P-value | <0.001 | <0.001 | <0.001 | <0.001 | <0.001 | <0.001 |
|  | AIC* | 683.6 | 695.0 | 695.3 | 683.6 | 695.0 | 695.3 |

Shown are results from linear mixed models with a random time slope and random intercept for subject, and including age, sex and education as covariates, and time, Tau-PET and an interaction between time and Tau-PET as predictors. Separate models were performed per cohort and per Tau-PET metric (TAU-SPEX or SUVr). We report the fixed effect of Tau-PET as the effect on cross-sectional MMSE, and the fixed effect of Tau-PET*Time as the effect on longitudinal MMSE.

*Note that AIC values are identical for the effect on cross-sectional MMSE and longitudinal MMSE within each cohort and within each Tau-PET metric as these come from the same linear mixed model.

**Supplementary Figure 1** Tau-negative individuals with TAU-SPEX values higher than 1%


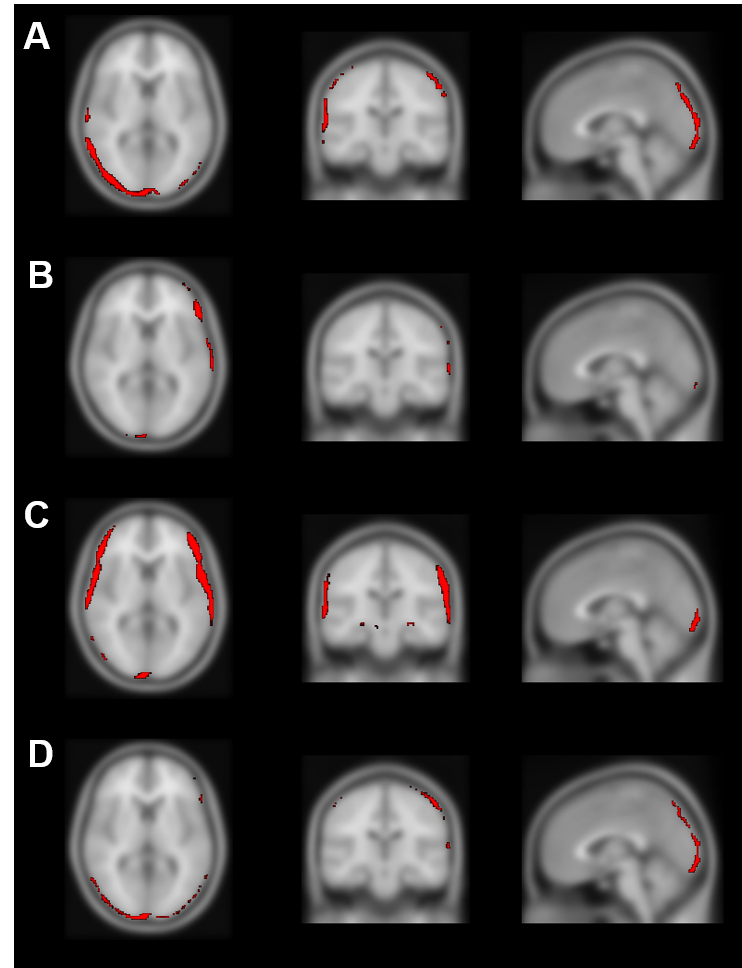


Since the threshold used to calculate TAU-SPEX is identical to the visual read threshold, we visually inspected scans that were visually tau-negative yet that had a TAU-SPEX value higher than 1% of which 4 scans are shown in this figure. These scans tended to show supra-threshold tracer retention in brain areas vulnerable for off-target binding, which affects TAU-SPEX (but not visual read).

**Supplementary Figure 2** TAU-SPEX and SUVr in relation to Tau-PET visual read

**
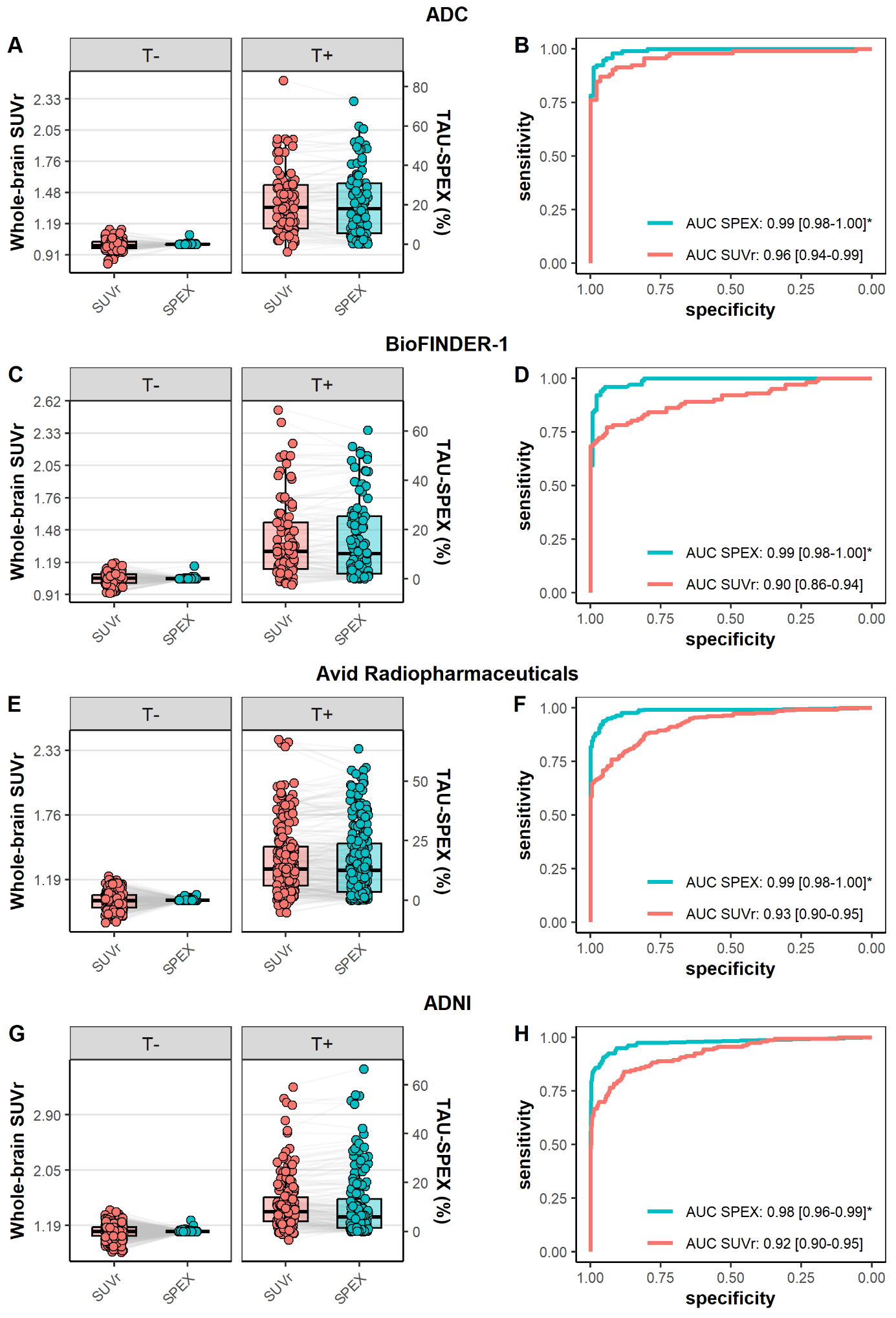
**

Shown in A, C, E and G are TAU-SPEX and SUVr values (double y-axes) in visual read tau-negative (T-) and visual read tau-positive (T+) participants in each cohort. To enable plotting TAU-SPEX and SUVr on the same axis, we first z-transformed each Tau-PET metric within each cohort. For interpretation purposes, we included raw y-axis scales instead of the z-transformed y-axis scales. The scale for SUVr is shown on the left, and the scale for TAU-SPEX is shown on the right. We linked data-points from the same participants using grey lines in order to visualize the reduced variance in TAU-SPEX within visual read tau-negative participants, and the maintained (or increased) variance in TAU-SPEX within visual read tau-positive participants. Shown in B, D, F and H are the Receiver Operating Characteristic (ROC) curves for Tau-PET visual read against TAU-SPEX and whole-brain SUVr.

**Supplementary Figure 3** Association of TAU-SPEX and SUVr with longitudinal performance on the MMSE


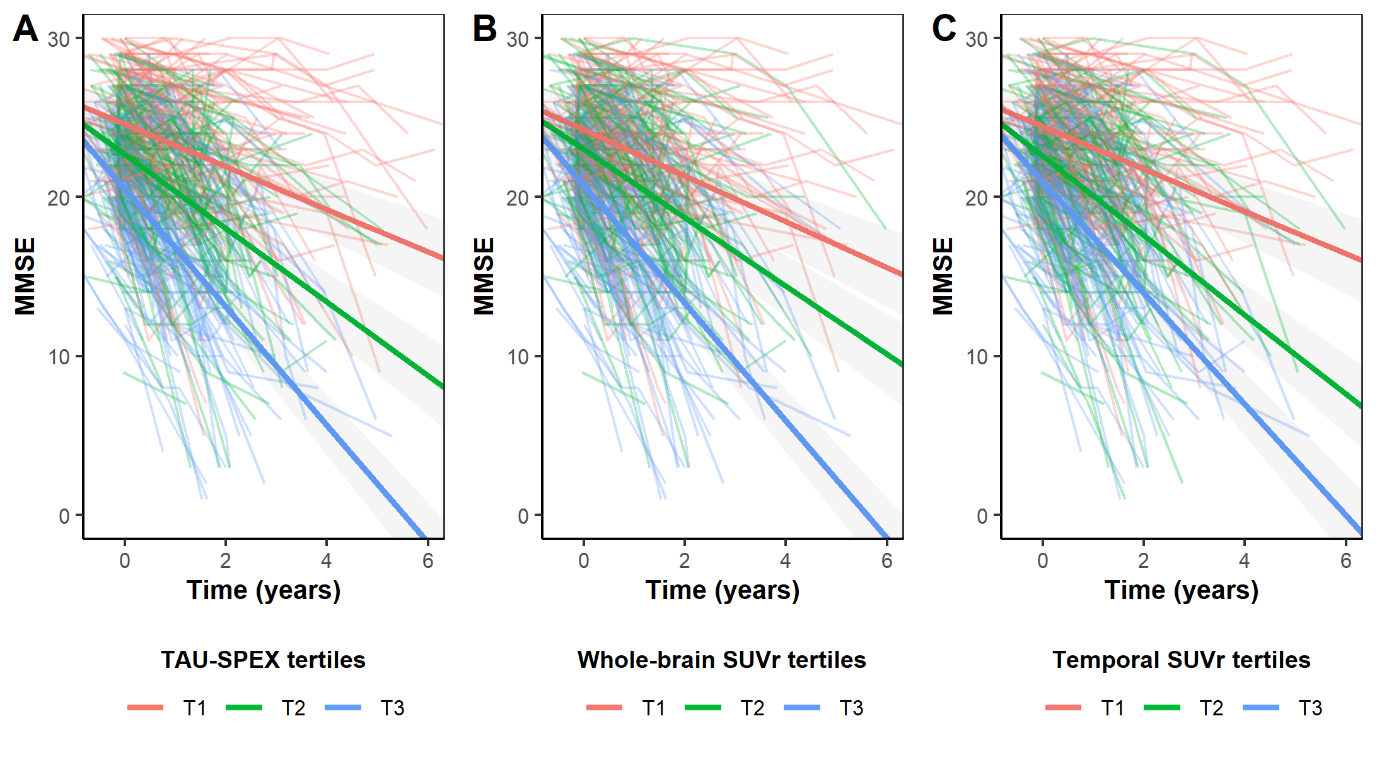


Shown are spaghetti plots visualizing the association between TAU-SPEX (A), whole-brain SUVr (B) and temporal SUVr (C) with longitudinal performance on the MMSE. For visualization purposes, we superimposed group-average lines derived from linear mixed models for participants in each TAU-SPEX or SUVr tertile.

**References**

Ossenkoppele, R., Rabinovici, G. D., Smith, R., Cho, H., Scholl, M., Strandberg, O., . . . Hansson, O. (2018). Discriminative Accuracy of [18F]flortaucipir Positron Emission Tomography for Alzheimer Disease vs Other Neurodegenerative Disorders. *JAMA, 320*(11), 1151-1162. doi:10.1001/jama.2018.12917

Petersen, R. C., Aisen, P. S., Beckett, L. A., Donohue, M. C., Gamst, A. C., Harvey, D. J., . . . Weiner, M. W. (2010). Alzheimer's Disease Neuroimaging Initiative (ADNI): clinical characterization. *Neurology, 74*(3), 201-209. doi:10.1212/WNL.0b013e3181cb3e25

Pontecorvo, M. J., Devous, M. D., Kennedy, I., Navitsky, M., Lu, M., Galante, N., . . . Mintun, M. A. (2019). A multicentre longitudinal study of flortaucipir (18F) in normal ageing, mild cognitive impairment and Alzheimer's disease dementia. *Brain, 142*(6), 1723-1735. doi:10.1093/brain/awz090

van der Flier, W. M., & Scheltens, P. (2018). Amsterdam Dementia Cohort: Performing Research to Optimize Care. *J Alzheimers Dis, 62*(3), 1091-1111. doi:10.3233/JAD-170850
